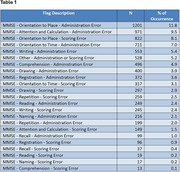# Identifying and Addressing Common Rater Errors in MMSE Administration and Scoring

**DOI:** 10.1002/alz70859_100258

**Published:** 2025-12-25

**Authors:** Sayaka Machizawa, Gila Barbati, Madison Soleil DeCapo, Andrei Iacob, Jessica Stenclik, Erica R. Appleman, Amanda Hackebeil

**Affiliations:** ^1^ Signant Health, Blue Bell, PA USA; ^2^ Signant Health, Iasi Romania

## Abstract

**Background:**

The Mini‐Mental State Examination (MMSE) is a widely used cognitive instrument that is validated and translated into many languages. In clinical trials, it is frequently utilized as an eligibility screening tool, though less commonly as an endpoint. Despite its broad use, administration and scoring errors are prevalent, stemming from factors such as differing scale and scoring guidelines, varying levels of rater experience, and inconsistent application of scoring criteria. This study explores common administration and scoring errors identified among site raters in multinational Alzheimer’s disease clinical trials.

**Method:**

Central reviews of MMSE assessments were conducted in two multinational Phase 3 Alzheimer’s disease trials, where the MMSE was administered during the screening visit. Trained, qualified, and calibrated central reviewers performed audio and data reviews of the MMSE assessments. Errors in administration and scoring were identified using “flags,” with detailed descriptions provided for each flagged error. Frequencies of flagged errors were calculated. Both studies utilized the same set of flags and the same training materials for the scale to ensure consistency.

**Result:**

A total of 10,203 MMSE assessments were reviewed, with 26.8% flagged for administration errors and 27.0% for scoring errors. The frequency of flagged errors is summarized in Table 1. The five most common errors, in descending order, were: administration errors in Orientation to Place (11.8%), administration errors in Attention and Calculation (9.7%), scoring errors in Orientation to Place (9.5%), administration errors in Orientation to Time (7.0%), and administration errors in Writing (5.4%).

**Conclusion:**

Although the MMSE is widely recognized and utilized by clinicians, our study revealed that errors in its administration and scoring are relatively common. Non‐standardized practices can introduce variability and noise into clinical trial data, potentially compromising study outcomes. Addressing these issues requires the implementation of robust data quality monitoring strategies, such as central reviews and blinded data analysis, to promptly identify and correct errors, prevent recurrence, and ensure consistent rater calibration. Additionally, incorporating these findings into rater training programs can effectively reduce common errors, ultimately improving the accuracy and overall quality of data in clinical trials.